# Eccrine Spiradenoma Arising from the Breast Skin

**DOI:** 10.1155/2015/615158

**Published:** 2015-07-07

**Authors:** Mark A. Benedict, Ugur Ozerdem

**Affiliations:** Department of Pathology, Yale University School of Medicine, New Haven, CT 06520, USA

## Abstract

Eccrine spiradenomas are uncommon, benign lesions, which are thought to originate from the eccrine sweat glands. They are common in young adults and are without a sex predilection. Here we report a case of eccrine spiradenoma of the breast skin in a 39-year-old woman who presented with a breast nodule for 10 years. It is crucial to take eccrine spiradenoma into consideration in superficial, well-circumscribed, breast skin/subcutaneous lesions. It is useful to recognize the two-cell populations constituting this tumor: small, dark, basaloid cells with hyperchromatic nuclei, which are immunoreactive for P63 and calponin, and larger cells with a pale nucleus, often near the center of the cluster (inner cells), which are immunoreactive for CK7 and CD117 (C-kit).

## 1. Introduction

Due to the proximity of the skin and subcutis to the breast, the possibility of a “breast mass” actually representing a dermatologic lesion should be considered, particularly if the proliferation does not look characteristically “mammary” in appearance [1, 2]. One such “breast mass” is eccrine spiradenoma of skin.

Eccrine spiradenomas (ES) are rare lesions, which are thought to originate from the eccrine sweat glands [3–6]. Herein we report a case of eccrine spiradenoma of the breast skin in a 39-year-old woman who has presented with a palpable breast nodule for 10 years.

## 2. Case Report

A 39-year-old woman presents with a history of a small lesion of her left upper outer quadrant breast for the past 10 years. Recently, the lesion has enlarged with increasing symptomatology. However, the lesion was not painful. Mammography and ultrasonographic imaging reveal 1.8 cm well-circumscribed, oval mass in the left breast at 1 o'clock immediately under the skin. The patient has no other past medical history involving the breast and reports no family history of breast cancer. No needle core biopsy or fine needle aspiration biopsy is performed for this superficial (1 mm below the skin) lesion. Excisional biopsy under local anesthesia has been recommended since this superficial, radiographically benign, symptomatic skin lesion has been amenable to excision with clear margins in one-step excisional biopsy for pathologic diagnosis and therapy.

**Figure 1:**
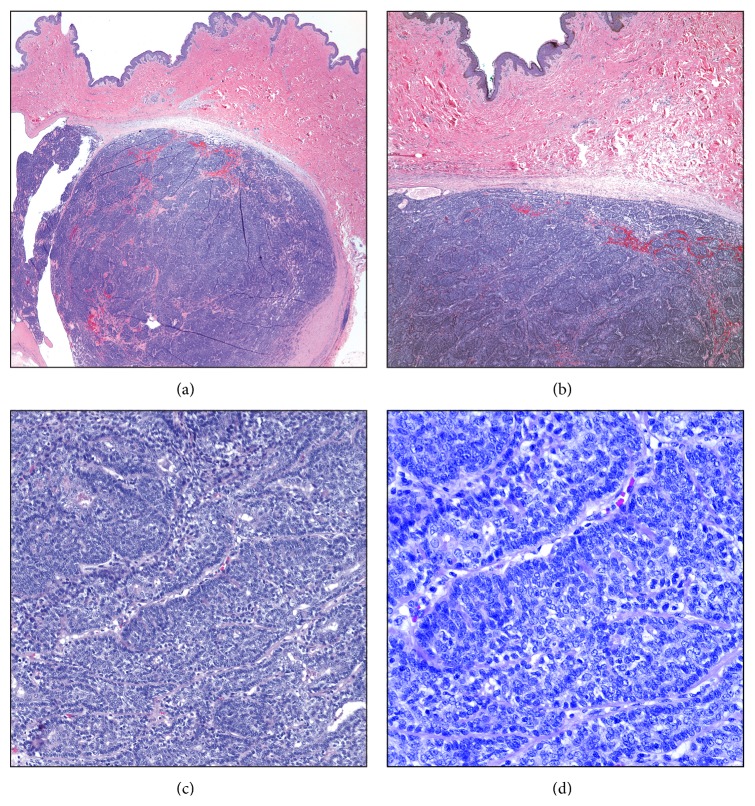
Eccrine spiradenoma of breast. The tumor is located at the interface of the deep dermis and superficial breast tissue, well-circumscribed with a thick fibrotic capsule (a and b). Two-cell populations within the tumor clusters are identified with dark-grey and light-grey nuclei being evident. The two-cell populations constituting this tumor: small dark and basaloid cells with hyperchromatic nuclei and larger cells with a pale nucleus, often near the center of the cluster.

**Figure 2:**
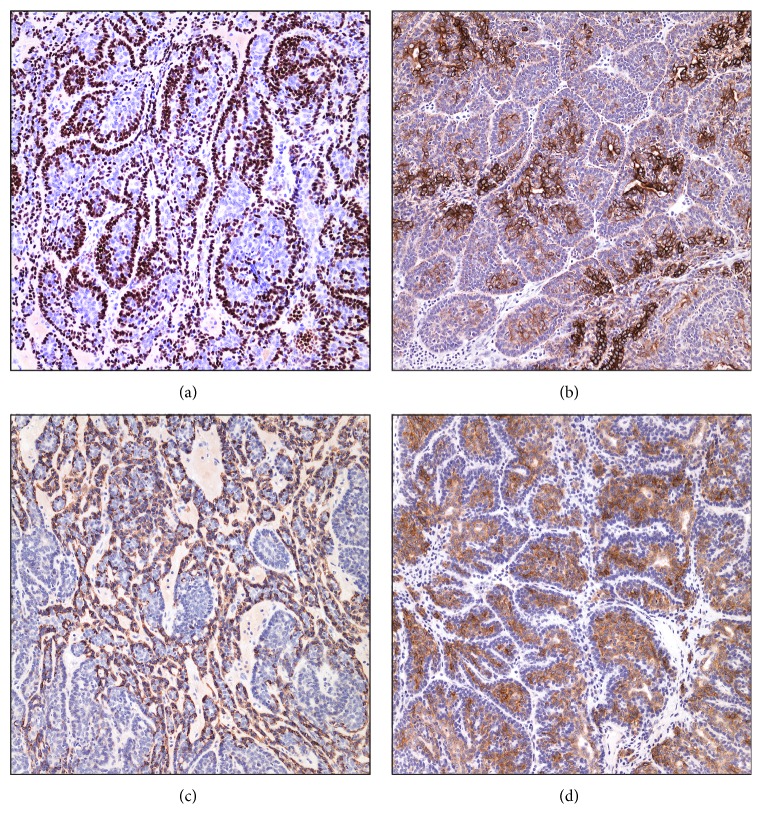
Immunoprofile of two-cell populations in eccrine spiradenoma. Basaloid cells are immunoreactive for P63 and calponin (a and c) and larger cells near the center of the clusters are immunoreactive for CK7 and CD117 (C-kit) (b and d). Both cell types are negative for ER, PR, AR, and HER2 expression (not shown).

An excisional biopsy of the left breast lesion has revealed a tan-white, well-circumscribed predominantly solid mass with no evidence of invasion into the surrounding tissue. Histologically, the tumor is located at the interface of the deep dermis and superficial breast tissue. Microscopically, the lesion has displayed mostly solid and focally cystic architecture. Two-cell populations within the tumor clusters are identified with dark-grey nuclei (basaloid cells) and with light-grey larger nuclei (inner cells) (Figure 1). Cytological atypia, necrosis, and mitoses are not appreciated. Scattered lymphocytes were seen amongst the tumor cell clusters as well as focal hyaline droplets in the stroma. These two- cell populations are immunoreactive for P63 and CK7, respectively. Most of the P63-immunoreactive basaloid cells also show immunoreactivity for calponin. CK7-immunoreactive inner cells display CD117 immunoreactivity (Figure 2). The tumor cells are quadruple-negative, that is, negative for ER, PR, AR, and HER2 on immunohistochemistry (not shown).

## 3. Discussion

Eccrine spiradenomas are benign tumors with malignant transformation being reported only on occasion. These tumors most commonly present as tender lesions and are typically solitary, intradermal, well-circumscribed, and firm. Most patients develop these lesions in their second to fourth decades. ES typically presents on the ventral aspect of the body with unusual involvement of the ear, eyelid, lip, and hand being reported [7, 8]. The few recurring lesions are consequent on incomplete excision.

It is crucial to take ES into consideration in superficial, well-circumscribed, breast skin lesions. It is useful to recognize the two-cell populations constituting this tumor: small, dark, basaloid cells with hyperchromatic nuclei, which are immunoreactive for P63 and calponin, and larger cells with a pale nucleus, often near the center of the cluster (inner cells), which are immunoreactive for CK7 and CD117 (C-kit).
